# Achieving a high coverage – the challenge of controlling HIV spread in heroin users

**DOI:** 10.1186/1477-7517-4-8

**Published:** 2007-02-15

**Authors:** Ming-qiang Li, Shui-shan Lee, Zhi-gao Gan, Yi Tan, Jin-Huai Meng, Ming-liang He

**Affiliations:** 1 Liuzhou Center for Disease Control and Prevention, Guangxi, China; 2 Stanley Ho Centre for Emerging Infectious Diseases, The Chinese University of Hong Kong, Hong Kong, China

## Abstract

In China, the national plan to open 1000 methadone clinics over a five-year period provides a unique opportunity to assess the impacts of harm reduction in a country with concentrated HIV epidemic amongst heroin users. To track the progress of this public health response, data were collected from the first methadone clinic in Liuzhou, Guangxi, a province with a high HIV prevalence. In the first 15 months of its operation, a cumulative total of 488 heroin users, 86% of which male, had joined the programme. The first dose of methadone was given efficiently at a median of 2 days after registration. Of the 240 heroin users attending the clinic in August 2006, 61% took methadone for four days or more each week. The number of active methadone users, however, leveled off at around 170 after the first two months, despite the availability of capacity to deliver more services. The reasons for this observation are: firstly, the provision of one single service that may not be convenient to all heroin users; and secondly, concerns of heroin users who may feel insecure to come forward. As broad  coverage is essential in ultimately reducing HIV risk, a low threshold  approach is crucial, which should be supported by the removal of  social obstacles and a refinement of the administrative procedures.

## Background

The epidemic of heroin addiction has fuelled the global spread of HIV, a phenomenon that is clearly visible in many parts of Asia [1]. The growth of this dual epidemic calls for the development of effective public health responses, which include the introduction of harm reduction measures targeting injection drug users and the provision of antiretroviral therapy to those infected according to clinical indications [2,3]. The use of opiod agonist substitution treatment has been proven to reduce injection, needles-sharing and HIV infection in various studies, and is now a standard recommendation both for the treatment of addiction and for HIV prevention and control [3,4]. Internationally, the expanded access of methadone maintenance treatment is prioritized, through the scaling up of harm reduction programmes in many countries. Though there is no lack of evidence in support of methadone maintenance [5], debates have continued because of the relative scarcity of fully evaluated programmes in developing countries.

There are lessons to be learned from the recent initiatives of China where the HIV spread in heroin users has taken root in some provinces, especially those bordering the Golden Triangle [1]. Of the estimated 650,000 persons living with HIV in the country, heroin users who shared needles accounted for 44.3% of the total [1]. Over the last year, harm reduction has been introduced as one of the key national intervention strategy. The national plan was to set up methadone clinics in 1000 sites over a five-year period [6]. The future of China's HIV epidemic obviously depends on how effective the country is in its operationalisation of the harm reduction strategy. Guangxi is one of the hardest hit provinces so far, with the HIV prevalence in heroin users in rural areas high at 25% [5]. Methadone treatment has been introduced as a public health programme in the province since about two years ago. To assess the progress of this new targeted population-based strategy, we reviewed the work of one of the first methadone clinics in the country.

## Methods

Liuzhou is the second largest city of Guangxi. The reported number of heroin users in the Liuzhou City is around 7000. The clinic is housed within the Skin and Sexually Transmitted Disease Clinic of the City's Centre for Disease Control. While the Clinic is not situated at the heart of the City, it's within reach (3 Km radius) from where most heroin users cumulate. The Clinic is staffed by 5 doctors, 1 counselor, 2 nurses, 2 pharmacists and other supporting administrative personnel. We reviewed the case records and workload statistics of the Clinic since the clinic's opening in May 2005. An unstructured interview of 10 randomly selected clients was conducted by two of the authors at the clinic. Approval was sought from the local health department. Ethical approval was obtained from the Ethics Committee of the Chinese University of Hong Kong.

## Findings

Overall, between 11 and 56 (mean = 35) new drug users each month registered at the Liuzhou Methadone Clinic since its opening. As of the end of August 2006, a cumulative total of 488 heroin users, 86% of which male, had joined the programme. Registration is required for joining the programme, with the following entry criteria: (a) heroin use for over one year; (b) age 20 or above; (c) resident of the city; and (d) having passed the physical checkup. Individual application is then submitted for official endorsement by the authorised office. The first dose of methadone is given at a median of 2 days after registration (range: 0 to 9 working days). Of the 240 heroin users attending the clinic in August 2006, 61% took methadone at least 4 days each week.

**Figure 1:**
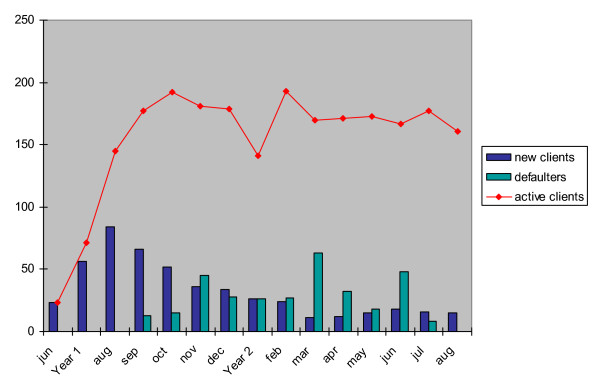
Registration of drugs users at a methadone clinic in Liuzhou, Guangxi.

The number of active methadone users has however leveled off at around 170 after the first two months (see figure 1). Despite a high number of heroin users in the city, new admission to the programme has not increased. Clearly the service of a clinic has not saturated, and there is adequate capacity to take in at least twice the current number of heroin users. Discussions with registered methadone users revealed a number of reasons. First of all, many heroin users may not be living in close proximity to the methadone clinic, and have therefore chosen not to travel long distances to access the service. According to the regulations, methadone must be taken under supervision on a daily basis at the clinic. Secondly, some heroin users did not feel comfortable in coming forward for treatment as they ran the risk of being arrested as drug taking is and has continued to be a criminal offence. It would take time for a common understanding to be developed by different government sectors on the role of methadone clinics at the field level. Thirdly, the strict criteria of admission also meant that only a fraction of the heroin users on the street are eligible for enrolment.

## Discussion

Against the background of an escalating HIV prevalence in heroin users around the world, it's reassuring to witness the establishment of substitution treatment in the world's most populous country. From a public health angle, there are lessons from the experiences in Liuzhou. Foremost, one key indicator in assessing the effectiveness of harm reduction is its coverage. Broad coverage serves two purposes: general reduction of risk behaviours [7]. and an alteration to the configuration of social networks of high risk-taking heroin users [8]. In Liuzhou, there're 1000 heroin users who have enrolled in a separate needle exchange programme. These, together with the current ones on methadone, account for some 20% of all heroin users in the city that have access to some forms of harm reduction service. Because of the low HIV prevalence in neighbouring Hong Kong, we use the latter's experience of having >60% heroin users in contact with the territory's methadone clinic network as a yardstick for assessing coverage [7]. With the plateau that has not been reached, it would take a long time before a reasonable coverage can be achieved in Liuzhou.

To improve coverage, substantial changes in social environment are needed, both in removing the obstacles and in facilitating the enrolment of heroin users. Setting up of small multiple clinics would be one strategy to promote coverage. With the functioning of just one clinic, the unmet needs cannot be managed effectively. The operation of multiple conveniently located methadone clinics or even out-reach services are means of solving the problem. The efforts required to set up multiple clinics in remote rural areas would likely be phenomenal. The existing programme falls short of a truly low threshold approach, the latter characterized by a combination of ease of access and the absence of obligatory requirement for staying on in the programme [9]. Restrictions imposed through the entrance criteria and high governmental expectation would easily discount the proportion of vulnerable community that could benefit from substitution treatment. Finally, it is clear that the establishment of methadone clinics reflects only the very first step towards the ultimate target of harm minimization on a population scale. Through this long process, means to improve coverage would be crucial.

## Competing interests

The author(s) declare that they have no competing interests.

## Authors' contributions

SL and MH conceptualized the study; YT collected data and conducted analysis; SL and TY conducted the interviews; ML, ZG and JM participated in data analysis and contributed to study design; SL prepared the manuscript and incorporated opinions from all others.
